# Interleukin-1 Beta rs16944 and rs1143634 and Interleukin-6 Receptor rs12083537 Single Nucleotide Polymorphisms as Potential Predictors of COVID-19 Severity

**DOI:** 10.3390/pathogens13100915

**Published:** 2024-10-21

**Authors:** Inas A. Ahmed, Taghrid G. Kharboush, Hiba S. Al-Amodi, Hala F. M. Kamel, Ehab Darwish, Asmaa Mosbeh, Hossam A. Galbt, Amal M. Abdel-Kareim, Shimaa Abdelsattar

**Affiliations:** 1 Department of Medical Biochemistry and Molecular Biology, Faculty of Medicine, Benha University, Benha 13518, Egypt; 2 Central Laboratory for Research, Faculty of Medicine, Benha University, Benha 13518, Egypt; 3 Department of Medical Biochemistry and Molecular Biology, Faculty of Medicine, Benha National University, El-Obour 11828, Egypt; 4 Department of Medical Microbiology and Immunology, Faculty of Medicine, Benha University, Benha 13518, Egypt; t.g.kharboush@gmail.com; 5 Department of Biochemistry, Faculty of Medicine, Umm Al-Qura University, P.O. Box 715, Makkah 21955, Saudi Arabia; hsamodi@uqu.edu.sa (H.S.A.-A.); hfkamel@uqu.edu.sa (H.F.M.K.); 6 Department of Medical Biochemistry and Molecular Biology, Faculty of Medicine, Ain Shams University, Cairo 11566, Egypt; 7 Department of Tropical Medicine, Faculty of Medicine, Zagazig University, Zagazig 44511, Egypt; ehab_drwish_123@yahoo.com; 8 Department of Internal Medicine, Faculty of Medicine, King Faisal University, AI-Ahsa 31982, Saudi Arabia; 9 Department of Pathology, National Liver Institute, Menoufia University, Menoufia 32511, Egypt; asmaa.abdelmaksoud@osumc.edu; 10 Department of Clinical Pathology, National Liver Institute, Menoufia University, Menoufia 32511, Egypt; hossamgalbt_2006@yahoo.com; 11 Department of Zoology, Faculty of Science, Benha University, Benha 13518, Egypt; amel.abdelkarim@fsc.bu.edu.eg; 12 Department of Clinical Biochemistry and Molecular Diagnostics, National Liver Institute, Menoufia University, Menoufia 32511, Egypt; shimaa.abdelsattar@liver.menofia.edu.eg

**Keywords:** COVID-19, SNPs, interleukin-6 receptor, interleukin-1 beta, genotype

## Abstract

Host genetic variation has been recognized as a key predictor of diverse clinical sequelae among severe acute respiratory syndrome coronavirus 2 (SARS-CoV-2)-infected patients. Insights into the link between the Interleukin-6 receptor (IL-6R) and Interleukin-1 beta (IL-1β) genetic variation and severe coronavirus disease 2019 (COVID-19) are crucial for developing new predictors and therapeutic targets. We aimed to investigate the association of IL-6R rs12083537, IL-1β rs16944, and IL-1β rs1143634 SNPs with the severity of COVID-19. Our study was conducted on 300 COVID-19-negative individuals (control group) and 299 COVID-19-positive cases, classified into mild, moderate, and severe subgroups. Analyses of IL-1β (rs16944, rs1143634) and IL-6R (rs12083537) SNPs’ genotypes were performed using qPCR genotyping assays. The IL-1β (rs16944) CC genotype and IL-6R (rs12083537) GG genotype were substantially related to COVID-19 severity, which was also associated with comorbidities and some laboratory parameters (*p* < 0.001). The IL-1β (rs1143634) TT genotype was found to be protective. Likewise, the IL-1β (rs16944) CC genotype was associated with increased mortality. IL-1β rs16944 and IL-6R rs12083537 SNPs are promising potential predictors of SARS-CoV-2 disease severity. Meanwhile, the rs1143634 SNP T allele was protective against severity and mortality risk.

## 1. Introduction

The existing pandemic of COVID-19 has created a global catastrophe, overwhelming healthcare facilities all over the world [1]. Multiple human coronavirus (CoV)-related emergencies have been discovered lately, including Severe Acute Respiratory Syndrome (SARS-CoV) in 2002, Middle East Respiratory Syndrome-CoV (MERS-CoV) in 2012, and most recently SARS-CoV-2, which emerged in 2019. In contrast to earlier coronaviruses, SARS-CoV-2 revealed a wider global dissemination, infecting more people than the SARS-CoV and MERS-CoV [2].

Various risk factors, such as old age and comorbidities, have markedly increased COVID-19 morbidity and death. While the majority of individuals affected by the illness experience recovery, mortality varies among individuals from different countries worldwide, raising a query concerning the preventive and risk factors of COVID-19 [3]. Numerous genetic investigations with varied geographical locations revealed significant genetic differences in the regions coding for the host–cell proteins with widely variable frequencies of alleles [4]. The consequences of SARS-CoV2 virus infection are reliant on a body response coordinated by the immune system [5].

The progression of SARS-CoV-2 infection from a mild illness to a more severe form has prompted researchers to speculate on the significance of genetic variation and exaggerated immune response in determining the susceptibility to and/or the severity of the disease [6]. SARS-CoV-2 has characteristics that contribute to its ethnic predominance, molecular structure, and pathophysiological effects on the body [7].

It was reported that cases with severe COVID-19 exhibited high levels of plasma cytokines and chemokines, reduced number of T lymphocyte subsets, and cytokine storm, pointing to the role of immune response dysregulation in the pathogenesis of COVID-19 [8]. The interleukin-1 (IL-1) family includes two distinct forms, IL-1α and IL-1β [9]. They are involved in regulating the immune system (innate and adaptive) and play a fundamental role in inflammation [10]. It was also reported that these two single nucleotide polymorphisms SNPs (rs16944 and rs1143634) located in the IL-1β gene were extensively studied for their association with the evolution of inflammatory disorders such as septic shock [11], congenital cytomegalovirus infection [12], and infection with H3N2, the seasonal influenza A virus [13].

IL-6 is a pleiotropic cytokine with a pro-inflammatory function [14]. Genetic variants of its receptor are implicated in the development of inflammatory and autoimmune diseases [15]. It was reported that SNP rs12083537 (A > G) is located in intron 1 of IL-6R andis closely linked with changes in the level of circulating C-reactive protein (CRP) [16]. IL-6 stimulates its target cells through a heterodimeric signaling complex including IL-6 ɑ-receptor (IL-6R) and the signal-transducing β-subunit glycoprotein 130 (gp130) [17]. Both membrane-bound IL-6R (mIL-6R) and soluble IL-6R (sIL-6R) forms can bind separately to IL-6 and induce gp130 homodimerization, which can activate the JAKs (Janus kinases) pathway [18]. IL-6 signaling-related indicators such as IL-6, sIL-6R, and soluble gp130 (sgp130) were discovered to be prognostic and diagnostic predictors of COVID-19 illness. Furthermore, models integrating IL-6 signaling factors were reported to be better than separate component analyses, regarding their diagnostic and prognostic efficacy in COVID-19 patients. Thus, IL-6 signaling markers may be valuable not only as indicators of severity, but also in developing novel treatment options in COVID-19 patients [19].

A multifactorial analysis may be able to highlight the potential risk variables that ifluence the pathogenesis and progress of SARS-CoV-2 infection. While Chinese communities have contributed to the majority of our understanding about the pathogenic behavior and the epidemiological pattern of COVID-19, comparatively less is known about other races [20].

Thereby, we aimed to investigate the association of IL-1β (rs16944 and rs1143634) and IL-6R (rs12083537) SNPs with COVID-19 severity among the Egyptian population.

## 2. Materials and Methods

A case–control study was conducted during the period from December 2021 to October 2022 on 599 Egyptian participants (300 healthy controls and 299 COVID-19 confirmed cases). Written informed consent was obtained from each participant before being enrolled in the study. The protocol of our study was approved by the Ethics Committee of the National Liver Institute, Menoufia University, and the Institutional Research Board number (IRBN) (protocol number 00426/2022). The current study was performed by a collaboration between the Central Laboratory for Research, Benha Faculty of Medicine, Benha University, and the National Liver Institute, Menoufia University.

Patients with a positive Reverse Transcription Polymerase Chain Reaction (RT-PCR) test of nasopharyngeal or oropharyngeal swabs for SARS-CoV-2 were involved in the current study. The controls were proved to be negative for COVID-19 using RT-PCR. Data retrieved from the patients’ electronic records included age, gender, and risky occupations (medical doctors, nurses, security guards, restaurant and delivery workers, bus drivers, housekeeping staff, teachers, and pharmacy or social workers frequently contacting cases in healthcare facilities). Additionally, comorbidities (diabetes mellitus, hypertension, cardiac disease, and bronchial asthma) and symptoms (cough, fever, sore throat, headache, myalgia, dyspnea, diarrhea, loss of smell and /or taste) were obtained electronically.

**Figure 1:**
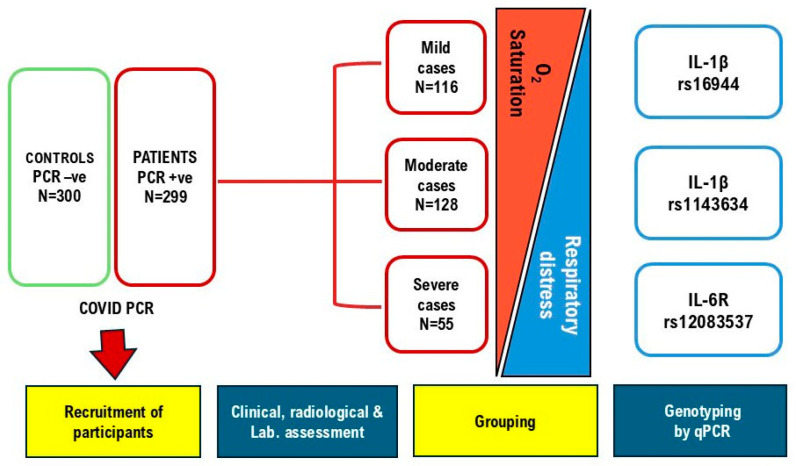
Study scheme.

All patients underwent a chest High-Resolution Computed Tomography (HRCT). Radiologists evaluated CT for scoring, in a clinical-data-blinded manner, according to a radiologic scoring system (CORAD) formerly defined by Bai et al. [21]. Patients were classified into three categories: mild, moderate, and severe. The following grading of severity was used: (1) mild: mild clinical symptoms, no pneumonia on lung CT; (2) moderate: fever, cough, and lung CT with pneumonia; and (3) severe: respiratory distress (respiratory rate > 30/min, oxygen saturation (O_2_ Sat) ≤ 93% at rest and/or ratio of arterial oxygen partial pressure to fractional inspired oxygen ≤ 300 mmHg (PaO2/FIO2) [22,23] (Figure 1).

### 2.1. Blood Sampling and Procedures

In total, 10 mL of venous blood was collected from each participant and distributed as follows: i. Samples for DNA extraction and genotyping: 3 mL of blood were evacuated into an ethylene diamine tetra–acetic acid (EDTA) tube and stored at −20 °C. ii. Samples for complete blood count (CBC) assay: 2 mL of blood were collected into another EDTA tube for CBC assay using a Sysmex XT-1800i hematology analyzer (Sysmex, Kobe, Japan). iii. Samples for biochemical assessment of C-reactive protein (CRP), ferritin, and Lactate dehydrogenase (LDH): 3 mL of blood were centrifuged in a plain test tube for serum separation. Cobas c 501 Auto analyzer (Roche, Mannheim, Germany) was used to assess for the CRP and Cobas e 601 Auto analyzers (Roche, Mannheim, Germany) was used for ferritin and LDH. iv. Sample for D dimer assessment using the Cobas 6000 analyzer (c501 module) (Roche, Mannheim, Germany): 2 mL blood were collected into sodium citrate vacutainer tubes.

### 2.2. Genotyping of IL-1β (rs16944, rs1143634) and IL-6R rs12083537 SNPs

Extraction of the Genomic DNA was performed for all the samples included in the study according to the manufacturer’s guidelines using a Gene JET TM whole blood Genomic DNA purification Mini kit from Thermo Fisher Scientific, Vilnius, European Union/Lithuania. DNA purity and quality were assessed using a Nanodrop One spectrophotometer (Thermo Fisher Scientific, Waltham, MA, USA). IL-1β rs16944 and rs1143634 SNPs and IL-6R rs12083537 were analyzed with qPCR in StepOne Plus system using a TaqMan genotyping assay kit for SNPs supplied from Thermo Fisher Scientific, Waltham, MA, USA, with catalog nos. C___1839943_10, C___9546517_10, and C__30997483_10 respectively.

The reaction mixture included 1 μL of the TaqMan SNP assay, TaqMan Genotyping master mix (5 μL), supplied by Applied Biosystems, Waltham, Massachusetts, USA, 500 ng of extracted genomic DNA, and a variable amount of nuclease-free water so that the DNA and water volume represented 9 μL in a total reaction volume of 15 μL. The following thermal conditions were applied: an initial denaturation at 95 °C for 10 min, followed by 40 cycles at 95 °C for 15 s (denaturing), and annealing/extension at 60 °C for 2 min.

The samples were tested in duplicates to confirm results, and 2 negative controls were included within each run, and StepOne Plus software v2.3 was used to generate the Genotypes of each SNP.

### 2.3. Statistical Analysis

The data collected was investigated by the use of Statistical Package for Social Science (IBM Corp., Released 2017. IBM SPSS Statistics for Windows, Version 25.0. Armonk, NY, USA: IBM Corp.). The expected deviancies from the Hardy–Weinberg equilibrium were assessed by utilizing the goodness of fit between the frequencies of the expected and observed genotypes. The association between two qualitative variables was tested by the use of a chi-square test. However, the relation between two qualitative variables when the expected count was less than 5 in more than 20% of cells was assessed using the Fisher Exact test. The significant statistical differences in the non-parametric variables between the two studied groups were examined using the Mann–Whitney Test. The Kruskal–Wallis Test was utilized to evaluate the statistical significance of the difference in a non-parametric variable between more than two study groups. Ordinal and binary logistic regression analyses were used for predicting the risk factors. The estimation of linkage disequilibrium (LD) and haplotypes was implemented by the use of the HaploView program (version 4.2), which uses expectation maximization (EM) algorithm. The *p*-value was considered significant if <0.05 at a confidence interval of 95%.

## 3. Results

The demographic and laboratory data of 299 COVID-19 patients versus 300 COVID-19 negative controls are presented in Table 1. The mean age was similar between the two groups; COVID-19 cases had a mean age of 48.4 ± 16.7 years, and controls had a mean age of 48.5 ± 15.91 years. There was an insignificant difference in the distribution of sex between the two groups. However, there was a significant difference in occupation, with a higher proportion of COVID-19 cases (42.8%) having a risky occupation compared to controls (17.3%). COVID-19 cases had significantly higher neutrophil/lymphocyte ratio (NLR), CRP, ferritin, LDH, significantly lower red blood cells (RBCs), hematocrit (HCT), platelets, and relative and absolute lymphocytic and neutrophilic counts. However, there were insignificant differences in hemoglobin (Hb), white blood cells (WBCs), and platelet/lymphocyte ratios (PLRs).

**Figure 2:**
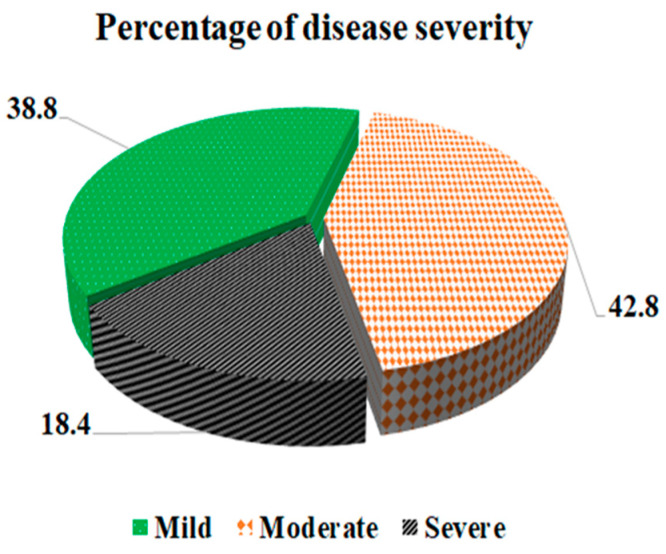
Pie chart for disease severity among cases of COVID–19.

Regarding the clinical data, the most common symptoms reported by COVID-19 cases were fever (80.3%) and cough (82.9%). Other common symptoms included sore throat (28.4%), loss of sense of smell (48.5%) and taste (32.1%), headache (51.5%), muscle ache (46.2%), and dyspnea (37.5%). Diarrhea (15.1%) symptoms were less frequently recorded. Approximately half of the cases (49.8%) had at least one comorbidity, such as hypertension (26.8%), diabetes mellitus (20.1%), heart disease (12.7%), and bronchial asthma (7.0%). The severity of COVID-19 cases varied, with 38.8% classified as mild, 42.8% as moderate, and 18.4% as severe (see Figure 2).

All studied SNPs were in Hardy–Weinberg equilibrium in the cases as well as in the controls. The COVID-19 cases were significantly associated with IL-6R rs12083537AG, GG genotypes, and the G allele. In addition, the COVID-19 cases were significantly associated with IL-1β rs16944 CC genotype and the C allele. On the other hand, the IL-1β rs1143634 CT, TT genotypes, and the T allele had lower frequencies in COVID-19 cases compared to controls and exhibited a protective effect against COVID-19, as shown in Table 2. Additionally, the association of rs12083537, rs16944 and rs1143634 with the different studied parameters are presented in Tables S1, S2,and S3 respectively.

According to NCBI, rs12083537 is located on chromosome 1 within the IL-6R gene, while rs16944 and rs1143634 are located on chromosome 2 within the IL-1β gene with 4477 bp (about 4.5 kb) apart. The rs16944–rs1143634 CC haplotype showed higher frequency in COVID-19 cases than controls, while the rs16944–rs1143634 CT, TT haplotype showed lower frequency in cases than controls, with a protective effect against COVID-19 development (see Table 3 and Figure S1).

Table 4 shows the rs12083537 GG genotype dominant and recessive models, and the G allele. The rs16944 CC genotype was substantially linked to increased COVID-19 severity grades. On the other hand, rs1143634 CT, TT genotypes, and the T allele showed significant protective effects against higher COVID-19 severity grades.

In Table 5, an ordinal regression analysis was used to predict severity-related factors. Older age, male gender, risky occupations, fever, headache, muscle ache, dyspnea, diarrhea, diabetes mellitus, heart disease, bronchial asthma, lower platelets, low absolute lymphocytic count, higher NLR, PLR, CRP, transferrin, LDH, and D dimer were associated with more-severe COVID-19. Meanwhile, loss of smell or taste were associated with less-severe COVID-19.

However, in Table 6, a multivariate analysis was conducted for the prediction of severity, and only older age, presence of comorbidities, high CRP, ferritin, LDH, low absolute lymphocytic count, rs12083537, and rs16944 polymorphisms were considered as independent higher-severity risk predictors. In contrast, rs1143634 polymorphism was considered an independent protective confounder against COVID-19 severity.

Out of 299 COVID-19 cases, 22 (7.4%) died. The recessive model of rs16944 was associated with a higher risk of mortality, while the rs1143634 TT genotype, T allele, and dominant model exhibited a significantly protective role against death. The rs12083537 was not associated with survival, as seen in Table 7.

## 4. Discussion

The immune response-associated genetic variation is assumed to impact COVID-19 disease susceptibility and severity [24]. Several studies have attempted to study the relationship between various gene polymorphisms and the severity of COVID-19 [25]. Limited information is available on various genes related to COVID-19 pathology, notably in Egypt [26]. Discovering host genetic pathways and gene polymorphisms that influence the infection risk and illness severity would considerably contribute to the progression of COVID-19 prophylactic and/or treatment strategies [4,27].

Cytokines are crucial to the pathophysiology of COVID-19. While some of them are helpful, others look deleterious, such as IL-6, especially in the setting of the cytokine storm [28]. IL-6, a pro-inflammatory cytokine that can be induced by IL-1β and a significant inducer of CRP, is consistently elevated in the serum of COVID-19 patients and strongly predicts poor prognosis [29,30]. A considerably higher amount of IL-1β in the bronchoalveolar lavage (BAL) fluid of COVID-19 patients was also linked with illness severity [31].

Thereby, the main perspective of our work was to study the association of three substantial gene polymorphisms (IL-6R rs12083537, IL-1β rs16944, and IL-1β rs1143634) with COVID-19 severity among Egyptian patients. We found a similarity in the mean age of the two groups. Additionally, an insignificant difference in the sex distribution with similar percentages of males and females was identified. However, there was a significant difference in occupation with a higher proportion of COVID-19 in cases (42.8%) having a risky occupation compared to the controls (17.3%). This agreed with the results of Pearce et al. [32], who recognized major differences in SARS-CoV-2 infection risk and mortality according to patients’ occupation. In their study, there was focus on the healthcare personnel and the risk was greater among the intensive care unit (ICU) workers in direct contact with COVID-19 patients. However, other occupations, especially those that entail social care of people or interaction with the public, may also be at a higher risk [32].

Regarding the laboratory data reported in our study, a significantly high NLR, CRP, LDH, and ferritin in addition to D dimer were reported among COVID-19 cases compared to controls. Our study also revealed that patients with older age, male gender, risky occupations, fever, headache, muscle ache, dyspnea, diarrhea, associated comorbidities (diabetes mellitus, hypertension, heart disease, and bronchial asthma), lower platelets, lymphocytes, and absolute lymphocyte count, and higher NLR, PLR, CRP, ferritin, LDH, and D dimer were related to the severity of COVID-19. However, loss of sense of smell or taste was associated with less-severe COVID-19. These results were parallel to Abdelsattar et al. [20], who identified significantly high levels of the inflammatory indicators, CRP, LDH, and ferritin, in addition to D dimer together with low neutrophils and lymphocytic counts. Additionally, they further suggested that such changes may reflect increased cytokine activity in severe cases of COVID-19. A higher NLR was detected in severe cases than in mild disease. Thus, NLR was suggested as a reliable indicator of COVID-19 disease severity [33,34]. Another study hypothesized that NLR is an inexpensive, robust, and available predictor of COVID-19 disease morbidity and mortality [35]. Similarly, Trofin, et al. [36] found that IL-6, CRP, ferritin, and LDH, in addition to D dimer, were raised in all the COVID-19 disease forms and D dimer could be used as a predictor of severity, while the LDH could foretell the SARS-CoV-2 variant. In line with our findings, published data from Italian patients with COVID-19 documented fever, dyspnea, and cough as the commonly associated symptoms. Furthermore, hypertension, type 2 diabetes, and ischemic heart disease were the most commonly associated comorbidities. In addition, around 20.3% of the patients were admitted to the ICU [37].

To our knowledge, this is one of the pioneer studies to scrutinize the IL-6R rs12083537 polymorphism with regard to SARS-CoV-2 infection. The principal finding in our study was an increased frequency of IL-6R rs12083537 GG genotypes among COVID-19 patients with a significant relationship to disease severity. Another study on an Amazonian population pointed to the presence of a consistent link between the polymorphisms of IL-6 and its receptor (IL-6R) with COVID-19 severity. This was probably attributable to greater expression of the genes associated with CC genotypes and their pro-inflammatory implications, supporting an earlier meta-analysis research linking them with global death rates, pneumonia, and immunobiological treatment procedures using IL-6 pathways [38]. IL-6R rs12083537 is positioned on chromosome 1 inside intron 1, and 2.9 kb far from exon 1 [14]. According to prior research on asthmatic patients, the rs12083537 SNP did not alter IL-6R gene transcription, since there was no significant link between rs12083537 and IL-6R mRNA levels. Nevertheless, rs12083537 might be recognized as a regulator for the levels of soluble IL-6R (sIL-6R) in the serum, with an impact on its function [39]. Another study in Han Chinese population patients with asthma revealed that IL-6R rs12083537 G is associated with poor lung function [40].

The genetic studies of critical COVID-19 cases revealed that genetic variations in the IL-6 inflammatory pathway are related to fatal diseases [41]. These findings lend support to a therapeutic approach that involves inhibiting IL-6 pathways in individuals with severe COVID-19. Treating ICU-admitted COVID-19 adult patients with the IL-6 receptor antagonists tocilizumab (TCZ) and sarilumab improved patients’ outcomes, including survival. Thus, IL-6 blocking is proclaimed as a promising therapeutic approach for COVID-induced Cytokine Release Syndrome (CRS) [42]. Respiratory illness in patients with Rheumatoid arthritis (RA) is a major contributor to morbidity and mortality [43]. However, RA patients having IL-6R rs12083537 GG genotype did not respond significantly to TCZ therapy [14].

Additionally, the COVID-19 cases included in the present study had IL-1β rs16944 CC genotype associated with risk to COVID-19 severity and mortality. The SNP rs16944 C/T in the IL-1β gene is linked to excessive IL-1β production with a greater risk of acquiring inflammatory disorders. IL-1β rs16944, the C/T genotype, showed some sort of vulnerability to develop COVID-19, whereas the T/T genotype was demonstrated to offer a protective function [44]. Another study had identified a significant relationship between the rs16944 A/G and SARS-CoV-2 risk. The AG variant genotype (AG vs. AA) showed an adjusted OR of 1.0 (95 percent CI = 1.770 (0.935–0.353), *p* = 0.078) when compared to the rs16944 AA genotype [45]. Another study on the risk of influenza A (H1N1) reported that both IL-1β and IL6 SNPs are significantly related to the disease severity, notably the rs16944, which is placed in the gene’s promoter area [46].

On the other hand, the present study reported that the IL-1β rs1143634 CT and TT genotypes had lower frequencies in COVID-19 cases compared to controls and exhibited a protective effect against COVID-19 mortality. Moreover, while examining the rs16944- rs1143634 haplotypes in cases and controls, we found that the CC haplotype has a higher frequency in cases than controls, with increased risk to COVID-19. However, the CT and TT haplotypes showed lower frequencies in cases than controls, with a protective effect against acquiring COVID-19. A study conducted in Ukraine found that the rs1143634 variation of the IL-1β gene, in COVID-19 patients, yielded the following genotype frequencies: CC–65.8%, CT–28.2%, and TT–6.0% [47]. The study results revealed that the patient group with the IL-1β gene T allele had greater WBCs counts (*p*  =  0.040), severe lymphopenia (*p*  =  0.007), and needed a significantly longer period on mechanical ventilators (*p*  =  0.049). Thus, IL-1β gene variants can be utilized as a predictor for evaluating the severity of COVID-19 pneumonia, which is opposite to our findings. Likewise, the authors recommended further study with a larger number of cases to validate their findings [47]. Contrary to our findings, another study on COVID-19 patients concluded that individuals who are carrying the G allele of the IL-1β rs1143634 SNP slowly progress from using mechanical ventilation systems or death outcomes [48].

Pulito-Cueto et al. [49] documented statistically insignificant differences in the haplotypic distribution of the IL-1β gene. Likewise, the rs1143634 and rs16944 SNP analysis revealed an insignificant association between GG, AG, and GA genotypes and COVID-19 severity. The discrepancy in the results noted in the literature could be due to differences in ethnicity and using different criteria to determine disease severity. Different age ranges, gender, associated comorbidities, and chosen sample sizes from the population are additional factors that may impact the reported results in various studies [50]. Further validations are needed to clarify this reported divergence.

In our study, the multivariate analysis indicated that only older age, presence of comorbidities, high CRP, ferritin, LDH, low absolute lymphocyte count, and rs12083537 and rs16944 polymorphisms were considered as independent higher-severity risk predictors. However, rs1143634 polymorphism was considered an independent protective confounder against COVID-19 severity. This was parallel to a study that reported age, gender, and comorbidities as common risk factors for COVID-19 severity. The authors also reported SNPs in numerous genes as a contributing genetic factor to severe COVID-19 [51].

The host genetic background encompassing the individual’s immune system may impact the relationship between the interleukin gene polymorphism variations with the COVID-19 prevalence and mortality rate [50].

## 5. Conclusions

The IL-1β rs1143634 SNP T allele was protective against severe COVID-19, while the IL-6R rs12083537 G allele and the IL-1β rs16944 C allele were markedly related to COVID-19 severity among Egyptians. Of note, the recessive model of rs16944 was associated with a higher risk of mortality, while the rs1143634 T allele exhibited a significantly protective role against death. However, the rs12083537 was not associated with survival. Genetic variation might impact COVID-19 severity and outcome. Hence, it should be considered for patient-targeted therapy.

## Figures and Tables

**Table 1:** Demographic and laboratory data among cases of COVID-19 compared to controls.

Demographic Data	COVID-19 N = 299	Control N = 300	*p*
**Age**			0.978
Mean ± SD.	48.4 ± 16.7	48.5 ± 15.91	
Median (Min.–Max.)	47 (13–85)	48 (20–85)	
**Sex**			0.838
Male N (%)	152 (50.8%)	150 (50.0%)	
Female N (%)	147 (49.2%)	150 (50.0%)	
**Occupation**			<0.001 *
Not risky N (%)	171 (57.2%)	248 (82.7%)	
Risky N (%)	128 (42.8%)	52 (17.3%)	
**Laboratory data**			
**Hb (g/dL)**			0.634
Mean ± SD.	12.5 ± 1.84	12.7 ± 1.83	
Median (Min.–Max.)	12.5 (7–16.6)	12.2 (8.9–16.3)	
**RBCs (×10^6^/mm^3^)**			<0.001 *
Mean ± SD.	5.04 ± 1.78	4.98 ± 0.42	
Median (Min.–Max.)	4.8 (2.5–14.8)	5 (4.2–5.9)	
**HCT (%)**			<0.001 *
Mean ± SD.	38 ± 7.42	42 ± 3.69	
Median (Min.–Max.)	40 (4.5–48)	41.5 (37–50)	
**Platelets (×10^3^/mm^3^)**			<0.001 *
Mean ± SD.	211 ± 83.5	312 ± 79.9	
Median (Min.–Max.)	206 (22–569)	322 (157–450)	
**WBCs (×10^3^/mm^3^)**			0.328
Mean ± SD.	6.64 ± 3.8	6.08 ± 1.29	
Median (Min.–Max.)	5.7 (2.1–28.1)	6.3 (4.1–9.5)	
**Lymphocytes (×10^3^/mm^3^)**			<0.001 *
Mean ± SD.	21.3 ± 11.3	31.1 ± 7.75	
Median (Min.–Max.)	19 (4–65)	30 (20–46)	
**Neutrophils (×10^3^/mm^3^)**			<0.001 *
Mean ± SD.	55.8 ± 19.2	61.9 ± 8.56	
Median (Min.–Max.)	54 (19.3–91)	65 (44–73)	
**NLR**			<0.001 *
Mean ± SD.	3.83 ± 3.61	2.18 ± 0.77	
Median (Min.–Max.)	2.67 (0.38–22.5)	2.1 (0.96–3.65)	
**PLR**			0.818
Mean ± SD.	12.8 ± 9.96	10.5 ± 3.46	
Median (Min.–Max.)	10.1 (1.26–56.9)	9.92 (3.93–20.9)	
**CRP (mg/L)**			<0.001 *
Mean ± SD.	79.4 ± 91.2	2.53 ± 1.35	
Median (Min.–Max.)	44.9 (0.45–528)	2.39 (0.29–5.34)	
**Ferritin (ng/mL)**			<0.001 *
Mean ± SD.	346 ± 310	18.1 ± 9.84	
Median (Min.–Max.)	200 (9–1119)	17 (2–45)	
**LDH (mg/L)**			<0.001 *
Mean ± SD.	431 ± 237	295 ± 54.7	
Median (Min.–Max.)	345 (53–1500)	285 (216–425)	
**D. Dimer (ng/mL)**			<0.001 *
Mean ± SD.	577 ± 633	142 ± 58.7	
Median (Min.–Max.)	320 (50–3500)	110 (50–300)	
**Absolute lymphocytic count (×10^3^/mm^3^)**			<0.001 *
Mean ± SD.	1347 ± 868	1892 ± 621	
Median (Min.–Max.)	1142 (135–4840)	1750 (820–3515)	
**Absolute neutrophil count (×10^3^/mm^3^)**			<0.001 *
Mean ± SD.	3948 ± 3358	3772 ± 991	
Median (Min.–Max.)	2970 (568–24447)	3569 (2156–5893)	

Hb: hemoglobin, RBCs: red blood cells, HCT: hematocrit test, WBCs: white blood cells, NLR: neutrophil-to-lymphocyte ratio, PLR: platelet-to-lymphocyte ratio, CRP: C-reactive protein, and LDH: lactate dehydrogenase. SD.: standard deviation, Min.: minimum, Max.: maximum, *p*: *p*-value, *: statistically significant at *p* < 0.05.

**Table 2:** Binary logistic regression analysis for prediction of COVID-19.

	COVID-19 N = 299	Control N = 300	*p*	OR (95% CI)
**rs12083537**				
**Multiplicative model**				
AA^®^	128 (42.8%)	198 (66.0%)		Reference
AG	141 (47.2%)	88 (29.3%)	<0.001 *	1.76 (1.42–2.19)
GG	30 (10.0%)	14 (4.7%)	<0.001 *	2.11 (1.40–3.17)
HWE	0.327	0.302		
**Dominant model**				
AA	128 (42.8%)	198 (66.0%)		Reference
AG + GG	171 (57.2%)	102 (34%)	<0.001 *	1.81 (1.48–2.22)
**Recessive model**				
AA + AG	269 (90%)	286 (95.3%)		Reference
GG	30 (10.0%)	14 (4.7%)	0.012 *	1.67 (1.12–2.49)
**Alleles**				
A	397 (66.4%)	484 (80.7%)		Reference
G	201 (33.6%)	116 (19.3%)	<0.001 *	1.59 (1.35–1.88)
**rs16944**				
**Multiplicative model**				
TT^®^	83 (27.8%)	103 (34.3%)		Reference
TC	150 (50.1%)	152 (50.7%)	0.278	1.14 (0.90–1.43)
CC	66 (22.1%)	45 (15.0%)	0.013 *	1.45 (1.08–1.96)
HWE	0.909	0.362		
**Dominant model**				
TT^®^	83 (27.8%)	103 (34.3%)		Reference
TC + CC	216 (72.2%)	197 (65.7%)	0.082	1.21 (0.98–1.51)
**Recessive model**				
TC + TC	233 (77.9%)	255 (85%)		Reference
CC	66 (22.1%)	45 (15.0%)	0.026 *	1.34 (1.04–1.74)
**Alleles**				
T	316 (52.8%)	358 (59.7%)		Reference
C	282 (47.2%)	242 (40.3%)	0.017 *	1.19 (1.03–1.37)
**rs1143634**				
**Multiplicative model**				
CC^®^	109 (36.5%)	32 (10.7%)		Reference
CT	135 (45.2%)	123 (41.0%)	<0.001 *	0.50 (0.38–0.66)
TT	55 (18.4%)	145 (48.3%)	<0.001 *	0.26 (0.19–0.35)
HWE	0.250	0.442		
**Dominant model**				
CC	109 (36.5%)	32 (10.7%)		Reference
CT + TT	190 (63.5%)	268 (89.3%)	<0.001 *	0.38 (0.29–0.49)
**Recessive model**				
CC + CT	244 (81.6%)	155 (51.7%)		Reference
TT	55 (18.4%)	145 (48.3%)	<0.001 *	0.41 (0.33–0.52)
**Alleles**				
C^®^	353 (59%)	187 (31.2%)		Reference
T	245 (41%)	413 (68.8%)	<0.001 *	0.49 (0.42–0.56)

OR: odds ratio, ^®^: reference group, HWE, Hardy–Weinberg equilibrium. N = Non-numerical data were expressed by using number (N) and (%). *p*: *p*-value, *: statistically significant at *p* < 0.05.

**Table 3:** The rs16944–rs1143634 haplotype prediction for COVID-19.

rs16944–rs1143634	COVID-19	Control	*p*	OR (95% CI)
CC	186.25 (31.1)	25.91 (4.3)	<0.001 *	10.022 (6.520–15.407)
CT	95.75 (16.0)	216.09 (36.1)	<0.001 *	0.339 (0.257–0.446)
TC	166.75 (27.9)	161.09 (26.8)	0.687	1.054 (0.817–1.358)
TT	149.25 (25.0)	196.91 (32.8)	0.003 *	0.681 (0.529–0.876)

*: statistically significant at *p* < 0.05.

**Table 4:** Association between severity of COVID-19 and gene polymorphism.

	Severity of COVID-19			*p*	OR (95% CI)
	Mild N = 116	Moderate N = 128	Severe N = 55		
**rs12083537**					
**Multiplicative model**					
AA	57 (49.1%)	53 (41.4%)	18 (32.7%)		Reference
AG	53 (45.7%)	65 (50.8%)	23 (41.8%)	0.284	1.16 (0.88–1.52)
GG	6 (5.2%)	10 (7.8%)	14 (25.5%)	<0.001 *	2.43 (1.54–3.83)
**Dominant model**					
AA	57 (49.1%)	53 (41.4%)	18 (32.7%)		Reference
AG + GG	59(50.9%)	75(58.6%)	37(67.3%)	0.038 *	1.31 (1.01–1.70)
**Recessive model**					
AA + AG	110(94.8%)	118(92.2%)	41(74.5%)		Reference
GG	6 (5.2%)	10 (7.8%)	14 (25.5%)	<0.001 *	2.25 (1.46–3.46)
**Alleles**					
A	167(72.0%)	171(66.8%)	59(53.6%)		Reference
G	65(28%)	85(33.2%)	51(46.4%)	0.001 *	1.36 (1.13–1.65)
**rs16944**					
**Multiplicative model**					
TT	39 (33.6%)	25 (19.5%)	19 (34.5%)		Reference
TC	64 (55.2%)	79 (61.7%)	7 (12.7%)	0.178	0.81 (0.60–1.10)
CC	13 (11.2%)	24 (18.8%)	29 (52.7%)	<0.001 *	2.16 (1.49–3.12)
**Dominant model**					
TT	39 (33.6%)	25 (19.5%)	19 (34.5%)		Reference
TC + CC	77(66.4%)	103 (80.5%)	36 (65.5%)	0.540	1.09 (0.82–1.46)
**Recessive model**					
TT + TC	103 (88.8%)	104 (81.3%)	26 (47.3%)		Reference
CC	13 (11.2%)	24 (18.8%)	29 (52.7%)	<0.001 *	2.46 (1.80–3.38)
**Alleles**					
T	142 (61.2%)	129 (50.4%)	45 (40.9%)		Reference
C	90 (38.8%)	127 (49.6%)	65 (59.1%)	<0.001 *	1.40 (1.17–1.68)
**rs1143634**					
**Multiplicative model**					
CC	24 (20.7%)	58 (45.3%)	27 (49.1%)		Reference
CT	51 (44.0%)	58 (45.3%)	26 (47.3%)	0.021 *	0.72 (0.54–0.95)
TT	41 (35.3%)	12 (9.4%)	2 (3.6%)	<0.001 *	0.27 (0.18–0.40)
**Dominant model**					
CC	24 (20.7%)	58 (45.3%)	27 (49.1%)		Reference
CT + TT	92 (79.3%)	70 (54.7%)	28 (50.9%)	<0.001 *	0.57 (0.43–0.74)
**Recessive model**					
CC + CT	75 (64.7%)	116 (90.6%)	53 (96.4%)		Reference
TT	41 (35.3%)	12 (9.4%)	2 (3.6%)	<0.001 *	0.32 (0.22–0.47)
**Alleles**					
C	99 (42.7%)	174 (68.0%)	80 (72.7%)		Reference
T	133 (57.3%)	82 (32.0%)	30 (27.3%)	<0.001 *	0.55 (0.46–0.67)

Non-numerical data were expressed by using number (N) and (%), *p*: *p*-value, *: statistically significant at *p* < 0.05.

**Table 5:** Association between severity of COVID-19 with other parameters.

	Severity of COVID-19			*p*	OR (95% CI)
	Mild (N = 116) N (%)	Moderate (N = 128) N (%)	Severe (N = 55) N (%)		
**Age (years)** Mean ± SD. Median (range.)	41.96 ± 15.04 37.5 (19–83)	50.17 ± 16.64 49 (13–85)	58 ± 14.68 62 (28–78)	<0.001 *	1.025 (1.017–1.033)
**Sex** Male Female	47 (40.5%) 69 (59.5%)	74 (57.8%) 54 (42.2%)	31(56.4%) 24 (43.6%)	0.015 *	0.729 (0.564–0.941)
**Occupation** Not risky Risky	74 (63.8%) 42 (36.2%)	79 (61.7%) 49 (38.3%)	18 (32.7%) 37 (67.3%)	0.001 *	1.557 (1.201–2.018)
Fever	73 (62.9%)	116 (90.6%)	51 (92.7%)	<0.001 *	2.793 (1.948–4.003)
Cough	95 (81.9%)	100 (78.1%)	53 (96.4%)	0.080	1.355 (0.964–1.905)
Sore throat	38 (32.8%)	30 (23.4%)	17 (30.9%)	0.494	0.906 (0.682–1.203)
Smell loss	69 (59.5%)	51 (39.8%)	25 (45.5%)	0.019 *	0.736 (0.57–0.951)
Taste loss	47 (40.5%)	40 (31.3%)	9 (16.4%)	0.002 *	0.641 (0.485–0.846)
Headache	31 (26.7%)	79 (61.7%)	44 (80%)	<0.001 *	2.688 (2.053–3.518)
Muscle ache	22 (19%)	80 (62.5%)	36 (65.5%)	<0.001 *	2.537 (1.944–3.31)
Dyspnea	5 (4.3%)	67 (52.3%)	40 (72.7%)	<0.001 *	4.215 (3.139–5.66)
Diarrhea	12 (10.3%)	16 (12.5%)	17 (30.9%)	0.002 *	1.763 (1.233–2.522)
Hypertension	9 (7.8%)	43 (33.6%)	28 (50.9%)	<0.001 *	2.599 (1.934–3.494)
Diabetes mellitus	15 (12.9%)	30 (23.4%)	15 (27.3%)	0.015 *	1.477 (1.078–2.025)
Heart disease	6 (5.2%)	21 (16.4%)	11 (20%)	0.003 *	1.791 (1.226–2.616)
Bronchial asthma	4 (3.4%)	7 (5.5%)	10 (18.2%)	0.002 *	2.256 (1.357–3.749)
Total Comorbidities	29 (25%)	81(63.3%)	39 (70.9%)	<0.001 *	2.428 (1.862–3.165)
Death	1 (0.9%)	6 (4.7%)	15 (27.3%)	<0.001 *	4.585 (2.651–7.928)
**Hb (g/dL)** Mean ± SD. Median (Min.–Max.)	12.51 ± 1.52 12.2 (7–16.6)	12.81 ± 2.02 12.85 (7–16)	11.88 ± 1.87 11.6 (7–15.3)	0.163	0.952 (0.889–1.02)
**RBCs (×10^6^/mm^3^)** Mean ± SD. Median (Min.–Max.)	5.02 ± 1.57 4.79 (2.5–14.8)	5.19 ± 1.94 4.8 (2.5–14.8)	4.73 ± 1.76 4.48 (2.5–14)	0.539	0.978 (0.911–1.05)
**HCT (%)** Mean ± SD. Median (Min.–Max.)	38.33 ± 6.03 39 (20.3–48)	38.2 ± 8.13 41 (4.5–47)	36.78 ± 8.29 40 (19.3–47)	0.270	0.99 (0.974–1.007)
**Platelets (×10^3^/mm^3^)** Mean ± SD. Median (Min.–Max.)	223.13 ± 76.04 209.5 (22–388)	209.19 ± 86.11 215 (22–569)	189.22 ± 88.99 188 (22–426)	0.013 *	0.998 (0.997–0.999)
**WBCs (×10^3^/mm^3^)** Mean ± SD. Median (Min.–Max.)	6.57 ± 2.95 5.9 (2.7–19)	6.48 ± 4.13 5.7 (2.1–28.1)	7.16 ± 4.57 5.5 (2.2–19.7)	0.454	1.013 (0.98–1.047)
**Lymphocytes (×10^3^/mm^3^)** Mean ± SD. Median (Min.–Max.)	26.11 ± 11.92 23 (5.6–65)	19.9 ± 10.26 17.65 (4–48)	14.38 ± 6.71 15 (4–37)	<0.001 *	0.956 (0.943–0.969)
**Neutrophils (×10^3^/mm^3^)** Mean ± SD. Median (Min.–Max.)	62.53 ± 14.07 66 (20.3–88)	45.09 ± 17.97 42 (19.3–90)	66.43 ± 19.4 73.4 (29.2–91)	0.316	0.997 (0.99–1.003)
**NLR** Mean ± SD. Median (Min.–Max.)	3.13 ± 2.09 2.68 (0.38–12.14)	3.24 ± 3.17 2.32(0.45–22.5)	6.68 ± 5.39 3.95(1.45–22.5)	<0.001 *	1.105 (1.063–1.148)
**PLR** Mean ± SD. Median (Min.–Max.)	10.28 ± 5.93 9.47 (1.26–34.14)	13.3 ± 10.43 10.72(2.32–56.9)	16.94 ± 13.59 12.24 (2.37–52.32)	<0.001 *	1.028 (1.014–1.041)
**CRP (mg/L)** Mean ± SD. Median (Min.–Max.)	22.72 ± 23.9 13.18 (0.45–100.9)	107 ± 104.54 70 (3–528)	134.55 ± 84.73 114(29–528)	<0.001 *	1.007 (1.005–1.008)
**Ferritin (ng/mL)** Mean ± SD. Median (Min.–Max.)	175.61 ± 187.88 120 (9–1116)	393.25 ± 314.18 257.5 (23–1116)	592.89 ± 303.35 612 (100–1119)	<0.001 *	1.002 (1.001–1.003)
**LDH (mg/L)** Mean ± SD. Median (Min.–Max.)	377.47 ± 176.08 336.5 (200–900)	444.04 ± 256.53 344.5 (53–1500)	511.42 ± 277.26 381 (215–1500)	<0.001 *	1.001 (1.000–1.002)
**D. Dimer (ng/mL)** Mean ± SD. Median (Min.–Max.)	475.07 ± 551.03 245 (50–2940)	579.1 ± 640.47 310 (50–3500)	787.24 ± 728.07 430(80–3500)	0.004 *	1.002 (1.001–1.004)
**Absolute lymphocytes count (×10^3^/mm^3^)** Mean ± SD. Median (Min.–Max.)	1616.67 ± 799.42 1457.5 (360–4500)	1265.69 ± 921.3 1052 (177–4840)	966.93 ± 694.87 779 (135–2820)	<0.001 *	0.998 (0.997–0.999)
**Absolute neutrophil count (×10^3^/mm^3^)** Mean ± SD. Median (Min.–Max.)	4259.69 ± 2573.64 3742 (729–16720)	3163.11 ± 3446.48 2493(568.4–24447)	5115.34 ± 4139.34 3075(1022–14972)	0.571	1.002 (0.999–0.003)

Hb: hemoglobin, RBCs: red blood cells, HCT: hematocrit test, WBCs: white blood cells, NLR: neutrophil-to-lymphocyte ratio, PLR: platelet-to-lymphocyte ratio, CRP: C-reactive protein, and LDH: lactate dehydrogenase. Ordinal regression analysis, *p*: *p*-value, *: statistically significant at *p* < 0.05.

**Table 6:** Ordinal regression analysis for prediction of COVID-19 severity.

	Univariate		Multivariate	
	*p*	OR (95% CI)	*p*	OR (95% CI)
Age (years)	<0.001 *	1.025 (1.017–1.033)	0.008 *	1.013 (1.003–1.023)
Male	0.015 *	0.729 (0.564–0.941)	0.659	0.934 (0.688–1.267)
Female				
Risky Occupation	0.001 *	1.557 (1.201–2.018)	0.282	1.215 (0.852–1.734)
Comorbidities	<0.001 *	2.428 (1.862–3.165)	0.001 *	1.772 (1.28–2.454)
NLR	<0.001 *	1.105 (1.063–1.148)	0.389	1.026 (0.968–1.088)
PLR	<0.001 *	1.028 (1.014–1.041)	0.855	1.002 (0.982–1.022)
CRP (mg/L)	<0.001 *	1.007 (1.005–1.008)	<0.001 *	1.006 (1.004–1.007)
Ferritin (ng/mL)	<0.001 *	1.002 (1.001–1.003)	<0.001 *	1.001 (1.001–1.002)
LDH (mg/L)	<0.001 *	1.001 (1.000–1.002)	0.046 *	1.002 (1.001–1.005)
D. Dimer (ng/mL)	0.004 *	1.002 (1.001–1.004)	0.677	1.002 (0.998–1.006)
Absolute lymphocyte count (×10^3^/mm^3^)	<0.001 *	0.998 (0.997–0.999)	0.026 *	0.995 (0.984–0.999)
rs12083537	<0.001 *	1.81 (1.48–2.22)	0.018 *	1.274 (1.155–1.449)
rs16944	0.026 *	1.34 (1.04–1.74)	0.024 *	1.413 (1.046–1.909)
rs1143634	<0.001 *	0.38 (0.29–0.49)	0.008 *	0.653 (0.477–0.895)

Ordinal regression analysis, *p*: *p*-value, *: statistically significant at *p* < 0.05.

**Table 7:** Logistic regression analysis for prediction of death among COVID-19 cases.

	Alive N = 277	Died N = 22	*p*	OR (95% CI)
**rs12083537**				
**Multiplicative model**				
AA^®^	119 (43.0%)	9 (40.9%)		Reference
AG	133 (48.0%)	8 (36.4%)	0.648	0.896 (0.561–1.433)
GG	25 (9.0%)	5 (22.7%)	0.114	1.659 (0.886–3.105)
**Dominant model**				
AA	119 (43.0%)	9 (40.9%)		Reference
AG + GG	158 (57.0%)	13 (59.1%)	0.851	1.042 (0.678–1.602)
**Recessive model**				
AA + AG	252 (91.0%)	17 (77.3%)		Reference
GG	25 (9.0%)	5 (22.7%)	0.059	1.753 (0.978–3.139)
**Alleles**				
A	371 (67.0%)	26 (59.1%)		Reference
G	183 (33.0%)	18 (40.9%)	0.291	1.181 (0.867–1.610)
**rs16944**				
**Multiplicative model**				
TT^®^	76 (27.4%)	7 (31.8%)		Reference
TC	144 (52.0%)	6 (27.3%)	0.167	0.688 (0.405–1.17)
CC	57 (20.6%)	9 (40.9%)	0.311	1.323 (0.77–2.272)
**Dominant model**				
TT^®^	76 (27.4%)	7 (31.8%)		Reference
TC + CC	201 (72.6%)	15 (68.2%)	0.661	0.902 (0.568–1.432)
**Recessive model**				
TC + TC	220 (79.4%)	13 (59.1%)		Reference
CC	57 (20.6%)	9 (40.9%)	0.035 *	1.639 (1.034–2.598)
**Alleles**				
T	296 (53.4%)	20 (45.5%)		Reference
C	258 (46.6%)	24 (54.5%)	0.390	1.169 (0.865–1.579)
**rs1143634**				
**Multiplicative model**				
CC^®^	96 (34.7%)	13 (59.1%)		Reference
CT	127 (45.8%)	8 (36.4%)	0.100	0.682 (0.433–1.076)
TT	54 (19.5%)	1 (4.5%)	0.035 *	0.401 (0.172–0.936)
**Dominant model**				
CC	96 (34.7%)	13 (59.1%)		Reference
CT + TT	181 (65.3%)	9 (40.9%)	0.026 *	0.611 (0.397–0.942)
**Recessive model**				
CC + CT	223 (80.5%)	21 (95.5%)		Reference
TT	54 (19.5%)	1 (4.5%)	0.083	0.483 (0.212–1.099)
**Alleles**				
C^®^	319 (57.6%)	34 (77.3%)		Reference
T	235 (42.4%)	10 (22.7%)	0.010 *	0.645 (0.461–0.902)

OR: odds ratio, ^®^: reference group. Non-numerical data were expressed using number (N) and (%). *p*: *p*-value, *: statistically significant at *p* < 0.05.

## Data Availability

Data are available upon request.

## Supplementary Materials

The following supporting information can be downloaded at: https://www.mdpi.com/article/10.3390/pathogens13100915/s1, Figure S1: Linkage disequilibrium between rs16944- rs1143634, among (A) cases and (B) control [52,53]. Table S1: Association between rs12083537 and studied parameters. Table S2: Association between rs16944 and studied parameters. Table S3: Association between rs1143634 and studied parameters.
