# Supplementary material for: Interleukin-1 Beta rs16944 and rs1143634 and Interleukin-6 Receptor rs12083537 Single Nucleotide Polymorphisms as Potential Predictors of COVID-19 Severity
Source: Pathogens. 2024 Oct 21;13(10):915. doi: 10.3390/pathogens13100915 (PMC11510688; doi:10.3390/pathogens13100915)
Supplement: Supplementary file 1 [file pathogens-13-00915-s001.zip › pathogens-3197503-supplementary.pdf]

Supplementary materials

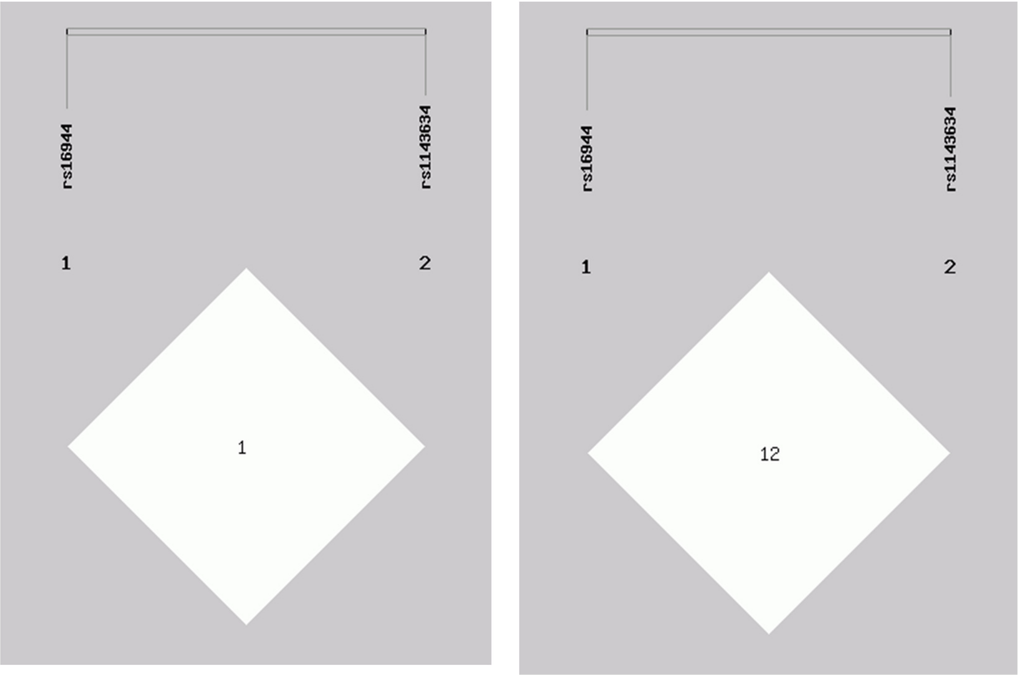

D′=0.010 | *D′*=0.127

A | **B**

Figure S1: Linkage disequilibrium between rs16944- rs1143634, among (A) cases and (B) controls.

Linkage disequilibrium (LD) is a measure of the non-random association of alleles at different loci within a population. *D′* varies between 0 (no disequilibrium) and 1 (maximum disequilibrium). Maximum disequilibrium occurs only when some haplotypes have a frequency 0; intermediate levels of disequilibrium imply recombination between markers or recurrent mutation [52]. For marker pair, the strength of association was measured; strong disequilibrium (D′ ⩾0.8), moderate (D′ 0.5-0.8), and weak (D′ 0-0.5) disequilibrium [53].

Table S1: Association between rs12083537 and studied parameters.

		rs12083537			p1	p2	p3	p4
		AA n=128	AG n=141	GG n=30				
**Male**		72(56.3%)	66(46.8%)	14(46.7%)				
**Females**		56(43.8%)	75(53.2%)	16(53.3%)	0.269	–	–	–
**Risky Occupation**		48(37.5%)	62(44%)	18(60%)				
**Fever**		101 (78.9%)	112 (79.4%)	27 (90%)	0.367	–	–	–
**Cough**		105 (82%)	116 (82.3%)	27 (90%)	0.555	–	–	–
**Sore throat**		38 (29.7%)	43 (30.5%)	4 (13.3%)	0.153	–	–	–
**Smell lost**		67 (52.3%)	63 (44.7%)	15 (50%)	0.448	–	–	–
**Taste loss**		43 (33.6%)	43 (30.5%)	10 (33.3%)	0.853	–	–	–
**Headache**		67 (52.3%)	68 (48.2%)	19 (63.3%)	0.313	–	–	–
**Muscle ache**		48 (37.5%)	69 (48.9%)	21 (70%)	0.004*	0.059	0.001*	0.036*
**Dyspnea**		43 (33.6%)	51 (36.2%)	18 (60%)	0.024*	0.658	0.007*	0.016*
**Diarrhea**		16 (12.5%)	23 (16.3%)	6 (20%)	0.496	–	–	–
**Hypertension**		26 (20.3%)	36 (25.5%)	18 (60%)	<0.001*	0.310	<0.001*	<0.001*
**DM**		19 (14.8%)	29 (20.6%)	12 (40%)	0.008*	0.221	0.002*	0.024*
**Heart disease**		11 (8.6%)	22 (15.6%)	5 (16.7%)	0.179	–	–	–
**Bronchial asthma**		3 (2.3%)	13 (9.2%)	5 (16.7%)	0.008*	0.017*	^{FE}0.007*	^{FE}0.321
**Comorbidity**		51 (39.8%)	77 (54.6%)	21 (70%)	0.004*	0.015*	0.003*	0.122
**Hb (g/dL)**	Mean ± SD.	12.7 ± 1.81	12.5 ± 1.82	11.9 ± 1.91	0.065	–	–	–
	Med. (Min. – Max.)	12.8 (7 – 16.6)	12.5 (7 – 16)	11.8 (7 – 16)				
**RBCS*10^6/mm^3**	Mean ± SD.	5.46 ± 2.30	4.79 ± 1.23	4.43 ± 0.69	0.001*	0.009*	0.001*	0.071
	Med. (Min. – Max.)	4.8 (2.5 – 14.8)	4.7 (2.5 – 14.8)	4.49 (2.5 – 5.6)				
**HCT(%)**	Mean ± SD.	38.2 ± 7.77	38 ± 7.14	37 ± 7.32	0.587	–	–	–
	Med. (Min. – Max.)	40 (4.5 – 48)	39.6 (19.3 – 47)	40.3 (20.3 – 45)				
**Platelets (*10^3/mm^3)**	Mean ± SD.	206 ± 81.2	219.4 ± 87.7	193 ± 69.6	0.139	–	–	–
	Med. (Min. – Max.)	202 (22 – 395)	215 (22 – 569)	175 (100 – 369)				
**WBCs (*10^3/mm^3)**	Mean ± SD.	6.59 ± 3.36	6.52 ± 3.86	7.39 ± 5.17	0.827	–	–	–
	Med. (Min. – Max.)	5.9 (2.6 – 24.5)	5.7 (2.1 – 28.1)	5.5 (2.2 – 19.7)				
**Lymphocytes (*10^3/mm^3)**	Mean ± SD.	21.5 ± 11.1	21.7 ± 11.7	18.5 ± 9.68	0.418	–	–	–
	Med. (Min. – Max.)	19 (5 – 65)	19 (4 – 65)	18.3 (5.9 – 48)				
**Neutrophils (*10^3/mm^3)**	Mean ± SD.	55.5 ± 18.6	55.1 ± 19.9	59.8 ± 19.2	0.457	–	–	–
	Med. (Min. – Max.)	55.3 (19.3 – 90)	54 (20.3 – 91)	54.5 (25 – 91)				
**NLR**	Mean ± SD.	3.59 ± 3.1	3.89 ± 4.01	4.56 ± 3.70	0.216	–	–	–
	Med. (Min. – Max.)	2.7 (0.38 – 17)	2.6(0.42 – 22.5)	3.51(0.92–15.2)				
**PLR**	Mean ± SD.	11.7 ± 8.34	13.6 ± 11.2	13.7 ± 10.2	0.481	–	–	–
	Med. (Min. – Max.)	9.48(1.26–52.3)	10.8(1.72–56.9)	10.6(3.19–48.8)				
**CRP (mg/L)**	Mean ± SD.	73.9 ± 101	79.9 ± 84.7	100 ± 76.9	0.053	–	–	–
	Med. (Min. – Max.)	33(0.45 – 528)	45(0.45 – 349)	91 (4 – 248)				
**Ferritin (ng/ml)**	Mean ± SD.	320 ± 291	334 ± 313	506 ± 336	0.014*	0.943	0.006*	0.005*
	Med. (Min. – Max.)	198 (14 – 1116)	186 (9 – 1119)	431 (43 – 1116)				
**LDH(mg/L)**	Mean ± SD.	441 ± 241	410 ± 216	483 ± 308	0.790	–	–	–
	Med. (Min. – Max.)	346(53–1500)	345(200–1500)	346(200–1500)				
**D. Dimer(ng/ml)**	Mean ± SD.	538 ± 562	567 ± 652	789 ± 789	0.210	–	–	–
	Med. (Min. – Max.)	338(80 – 3500)	300(50 – 3500)	480(80 – 3500)				
**Absolute lymphocytic count(*10^3/mm^3)**	Mean ± SD.	1352 ± 818	1370 ± 933	1220 ± 771	0.662	–	–	–
	Med. (Min. – Max.)	1177 (246 – 4500)	1142 (135 – 4840)	1043 (176 – 3198)				
**Absolute neutrophil count(*10^3/mm^3)**	Mean ± SD.	3750 ± 2531	3905 ± 3633	4995 ± 4775	0.673	–	–	–
	Med. (Min. – Max.)	3104 (589 – 13050)	2906 (568 – 24447)	2780 (750 – 16720)				

Hb: Hemoglobin, RBC: red blood cells, HCT: hematocrit test, WBC: White blood cells, NLR: Neutrophil-to-lymphocyte ratio, PLR: Platelet-to-Lymphocyte Ratio, CRP: C-reactive protein, and LDH: Lactate dehydrogenase. SD.: Standard deviation, Min.: Minimum, Max.: Maximum, Med.: Median, p1: Comparing AA, AG and GG. p2: Comparing AA and AG. p3: Comparing AA and GG. p4: Comparing AG and GG, *: Significant.

Table S2: Association between rs16944 and studied parameters.

		rs16944 TT n=83	rs16944 TC n=150	rs16944 CC n=66	p1	p2	p3	p4
**Male**		47(56.6%)	70(46.7%)	35(53%)	0.319	–	–	–
**Female**		36(43.4%)	80(53.3%)	31(47%)				
**Risky Occupation**		34(41%)	63(42%)	31(47%)				
**Fever**		67(80.7%)	118(78.7%)	55(83.3%)	0.724	–	–	–
**Cough**		70(84.3%)	120(80%)	58(87.9%)	0.338	–	–	–
**Sore throat**		26(31.3%)	40(26.7%)	19(28.8%)	0.750	–	–	–
**Smell lost**		45(54.2%)	69(46%)	31(47%)	0.467	–	–	–
**Taste loss**		23(27.7%)	52(34.7%)	21(31.8%)	0.552	–	–	–
**Headache**		45(54.2%)	69(46%)	40(60.6%)	0.119	–	–	–
**Muscle ache**		31(37.3%)	71(47.3%)	36(54.5%)	0.103	–	–	–
**Dyspnea**		26(31.3%)	47(31.3%)	39(59.1%)	<0.001*	0.999	0.001*	<0.001*
**Diarrhea**		12(14.5%)	19(12.7%)	14(21.2%)	0.266	–	–	–
**Hypertension**		14(16.9%)	37(24.7%)	29(43.9%)	0.001*	0.168	<0.001*	0.005*
**DM**		9(10.8%)	29(19.3%)	22(33.3%)	0.003*	0.093	0.001*	0.026*
**Heart disease**		8(9.6%)	18(12%)	12(18.2%)	0.279	–	–	–
**Bronchial asthma**		5(6%)	9(6%)	7(10.6%)	0.435	–	–	–
**Comorbidity**		28(33.7%)	78(52%)	43(65.2%)	0.001*	0.007*	<0.001*	0.073
**Hb (g/dL)**	Mean ± SD.	12.8 ± 1.83	12.6 ± 1.76	12 ± 1.94	0.017*	0.389	0.006*	0.022*
	Med. (Min. – Max.)	12.9 (7 – 16.6)	12.6 (7 – 16)	11.8 (7 – 16)				
**RBCS(*10^6/mm^3)**	Mean ± SD.	5.38 ± 2.22	4.91 ± 1.43	4.92 ± 1.83	0.146	–	–	–
	Med. (Min. – Max.)	4.8 (2.5 – 14.8)	4.8 (2.5 – 14.8)	4.7 (2.5 – 14)				
**HCT(%)**	Mean ± SD.	39 ± 7.49	38.3 ± 6.6	36 ± 8.72	0.042*	0.219	0.012*	0.045*
	Med. (Min. – Max.)	41 (4.5 – 48)	40 (19.3 – 47)	39 (4.51 – 46)				
**Platelets (*10^3/mm^3)**	Mean ± SD.	204 ± 87.5	218 ± 79.7	205 ± 86.8	0.450	–	–	–
	Med. (Min. – Max.)	200 (22 – 395)	215 (43 – 569)	194 (22 – 426)				
**WBCs(*10^3/mm^3)**	Mean ± SD.	7.07 ± 3.2	6.47 ± 3.92	6.48 ± 4.24	0.039*	0.028*	0.023*	0.610
	Med. (Min. – Max.)	6.4 (2.1 – 17)	5.6 (2.4 – 28.1)	5.7 (2.2 – 24.5)				
**Lymphocytes (*10^3/mm^3)**	Mean ± SD.	21.6 ± 12.5	21.9 ± 11.1	19.6 ± 9.9	0.284	–	–	–
	Med. (Min. – Max.)	19.3 (5 – 65)	19 (4 – 65)	17.3 (5 – 50)				
**Neutrophils (*10^3/mm^3)**	Mean ± SD.	58.1 ± 18.7	53.8 ± 19.1	57.4 ± 20	0.207	–	–	–
	Med. (Min. – Max.)	61 (20 – 90)	46.5 (20.3 – 91)	55 (19.3 – 91)				
**NLR**	Mean ± SD.	4.13 ± 3.80	3.56 ± 3.55	4.08 ± 3.54	0.118	–	–	–
	Med. (Min. – Max.)	2.71(0.38 – 17)	2.4(0.42 – 22.5)	2.97(0.48–17.6)				
**PLR**	Mean ± SD.	12.2 ± 10.1	13.2 ± 10.5	12.7 ± 8.52	0.351	–	–	–
	Med. (Min. – Max.)	9.21(2.32–52.3)	10.8(1.72–56.9)	10.3(1.26–48.8)				
**CRP (mg/L)**	Mean ± SD.	84.9 ± 119	71.1 ± 79.1	91.1 ± 75.1	0.027*	0.880	0.029*	0.010*
	Med. (Min. – Max.)	33 (0.45 – 528)	42.9(0.45–297)	83.5(1.2 – 297)				
**Ferritin (ng/ml)**	Mean ± SD.	344 ± 308	300 ± 288	452 ± 336	0.001*	0.132	0.045*	<0.001*
	Med. (Min. – Max.)	203(14–1116)	174(9–1116)	383(30–1119)				
**LDH (mg/L)**	Mean ± SD.	466 ± 274	399 ± 218	458 ± 223	0.076	–	–	–
	Med. (Min. – Max.)	350(200–1500)	329(53–1500)	348(200 – 890)				
**D. Dimer(ng/ml)**	Mean ± SD.	551 ± 588	553 ± 653	663 ± 642	0.072	–	–	–
	Med. (Min. – Max.)	320(50 – 3500)	250(50 – 3500)	450(100–3500)				
**Absolute lymphocytic count(*10^3/mm^3)**	Mean ± SD.	1424 ± 802	1369 ± 929	1199 ± 798	0.108	–	–	–
	Med. (Min. – Max.)	1333 (246 – 3640)	1138 (177 – 4840)	985 (135 – 3708)				
**Absolute neutrophil count(*10^3/mm^3)**	Mean ± SD.	4274 ± 2876	3808 ± 3664	3854 ± 3208	0.028*	0.009*	0.060	0.746
	Med. (Min. – Max.)	3366 (729 – 14696)	2808 (568 – 24447)	2873 (589 – 14972)				

Hb: Hemoglobin, RBC: red blood cells, HCT: hematocrit test, WBC: White blood cells, NLR: Neutrophil-to-lymphocyte ratio, PLR: Platelet-to-Lymphocyte Ratio, CRP: C-reactive protein, and LDH: Lactate dehydrogenase. SD.: Standard deviation, Min.: Minimum, Max.: Maximum, Med.: Median, p1: Comparing TT, TC, and CC. p2: Comparing TT and TC. p3: Comparing TT and CC. p4: Comparing TC and CC, *: Significant.

Table S3: Association between rs1143634 and studied parameters.

		rs1143634						
		CC n=109	CT n=135	TT n=55	p1	p2	p3	p4
**Male**		61(56%)	67(49.6%)	24(43.6%)				
**Female**		48(44%)	68(50.4%)	31(56.4%)	0.306	–	–	–
**Risky Occupation**		49(45%)	56(41.5%)	23(41.8%)				
**Fever**		98(89.9%)	107(79.3%)	35(63.6%)	<0.001*	0.024*	<0.001*	0.025*
**Cough**		90(82.6%)	113(83.7%)	45(81.8%)	0.944	–	–	–
**Sore throat**		29(26.6%)	46(34.1%)	10(18.2%)	0.077	–	–	–
**Smell lost**		47(43.1%)	65(48.1%)	33(60%)	0.124	–	–	–
**Taste loss**		32(29.4%)	44(32.6%)	20(36.4%)	0.654	–	–	–
**Headache**		69(63.3%)	69(51.1%)	16(29.1%)	<0.001*	0.056	<0.001*	0.006*
**Muscle ache**		58(53.2%)	64(47.4%)	16(29.1%)	0.013*	0.367	0.003*	0.020*
**Dyspnea**		59(54.1%)	44(32.6%)	9(16.4%)	<0.001*	0.001*	<0.001*	0.024*
**Diarrhea**		21(19.3%)	21(15.6%)	3(5.5%)	0.064	–	–	–
**Hypertension**		35(32.1%)	37(27.4%)	8(14.5%)	0.055	–	–	–
**DM**		22(20.2%)	30(22.2%)	8(14.5%)	0.487	–	–	–
**Heart disease**		17(15.6%)	16(11.9%)	5(9.1%)	0.459	–	–	–
**Bronchial asthma**		10(9.2%)	9(6.7%)	2(3.6%)	0.414	–	–	–
**Comorbidity**		60(55%)	71(52.6%)	18(32.7%)	0.018*	0.702	0.007*	0.013*
**Hb (g/dL)**	Mean ± SD.	12.6 ± 1.86	12.5 ± 1.94	12.5 ± 1.51	0.768	–	–	–
	Med. (Min. – Max.)	12.6 (7 – 16)	12.7 (7 – 16)	12.1(10.3–16.6)				
**RBCS(*10^6/mm^3)**	Mean ± SD.	5.06 ± 1.61	4.98 ± 1.74	5.15 ± 2.17	0.516	–	–	–
	Med. (Min. – Max.)	4.8(2.5 – 11.9)	4.8(2.5 – 14.8)	4.73(3.8 – 14.8)				
**HCT(%)**	Mean ± SD.	37.8 ± 7.33	37.89 ± 7.85	38.62 ± 6.55	0.834	–	–	–
	Med. (Min. – Max.)	39.6(4.51 – 47)	40 (4.5 – 48)	40 (20.3 – 48)				
**Platelets (*10^3/mm^3)**	Mean ± SD.	217 ± 88	205 ± 83.3	212 ± 74.9	0.379	–	–	–
	Med. (Min. – Max.)	220 (22 – 569)	200 (22 – 569)	209 (22 – 395)				
**WBCs(*10^3/mm^3)**	Mean ± SD.	6.93 ± 4.27	6.46 ± 3.55	6.49 ± 3.45	0.829	–	–	–
	Med. (Min. – Max.)	5.9 (2.1 – 28.1)	5.7 (2.4 – 28.1)	5.5 (2.2 – 19)				
**Lymphocytes (*10^3/mm^3)**	Mean ± SD.	19.6 ± 11	21.5 ± 10.3	24 ± 13.4	0.053	–	–	–
	Med. (Min. – Max.)	18 (4 – 65)	19 (4 – 55)	21 (5.6 – 65)				
**Neutrophils (*10^3/mm^3)**	Mean ± SD.	55.1 ± 20.5	55 ± 18.8	59.2 ± 17.6	0.347	–	–	–
	Med. (Min. – Max.)	47 (19.3 – 91)	54 (20 – 91)	63 (25 – 89.1)				
**NLR**	Mean ± SD.	4.29 ± 4.17	3.48 ± 3.18	3.77 ± 3.37	0.542	–	–	–
	Med. (Min. – Max.)	2.68(0.38–22.5)	2.6(0.45–22.5)	2.91(0.42–14.9)				
**PLR**	Mean ± SD.	14.6 ± 12	11.8 ± 8.69	11.7 ± 7.82	0.220	–	–	–
	Med. (Min. – Max.)	10.1(2.32–56.9)	10.3 (2 – 56.9)	9.42(1.26–34.1)				
**CRP (mg/L)**	Mean ± SD.	94 ± 99.3	79.6 ± 83.1	49.8 ± 87.9	<0.001*	0.220	<0.001*	0.001*
	Med. (Min. – Max.)	70 (1.2 – 528)	48 (0.6 – 297)	18(0.45 – 528)				
**Ferritin (ng/ml)**	Mean ± SD.	418 ± 323	356 ± 317	176 ± 177	<0.001*	0.026*	<0.001*	<0.001*
	Med. (Min. – Max.)	297 (30 – 1116)	190 (9 – 1119)	113 (9 – 830)				
**LDH(mg/L)**	Mean ± SD.	502 ± 287	406 ± 205	350 ± 153	0.006*	0.014*	0.003*	0.286
	Med. (Min. – Max.)	352(200–1500)	333(53 – 900)	333(200 – 890)				
**D. Dimer(ng/ml)**	Mean ± SD.	652 ± 678	632 ± 673	293 ± 250	0.003*	0.570	0.001*	0.003*
	Med. (Min. – Max.)	400(80 – 3500)	400(50 – 3500)	200(80 – 1210)				
**Absolute lymphocytic count(*10^3/mm^3)**	Mean ± SD.	1248 ± 797	1389 ± 927	1440 ± 852	0.243	–	–	–
	Med. (Min. – Max.)	1083 (177 – 3708)	1142 (135 – 4840)	1305 (168 – 4500)				
**Absolute neutrophil count(*10^3/mm^3)**	Mean ± SD.	4193 ± 3809	3693 ± 3020	4087 ± 3206	0.568	–	–	–
	Med. (Min. – Max.)	3053 (568 – 24447)	2829 (609 – 24447)	3366 (729 – 16720)				

Hb: Hemoglobin, RBC: red blood cells, HCT: hematocrit test, WBC: White blood cells, NLR: Neutrophil-to-lymphocyte ratio, PLR: Platelet-to-Lymphocyte Ratio, CRP: C-reactive protein, and LDH: Lactate dehydrogenase. SD.: Standard deviation, Min.: Minimum, Max.: Maximum, Med.: Median,, p1: Comparing CC, CT, and TT. p2: Comparing CC and CT; p3: Comparing CC and TT. p4: Comparing CT and TT, *: Significant.
